# Long Non-coding RNA NEAT1: A Novel Target for Diagnosis and Therapy in Human Tumors

**DOI:** 10.3389/fgene.2018.00471

**Published:** 2018-10-15

**Authors:** Peixin Dong, Ying Xiong, Junming Yue, Sharon J. B. Hanley, Noriko Kobayashi, Yukiharu Todo, Hidemichi Watari

**Affiliations:** ^1^Department of Obstetrics and Gynecology, Hokkaido University School of Medicine, Hokkaido University, Sapporo, Japan; ^2^State Key Laboratory of Oncology in South China, Department of Gynecology, Sun Yat-sen University Cancer Center, Guangzhou, China; ^3^Department of Pathology and Laboratory Medicine, University of Tennessee Health Science Center, Memphis, TN, United States; ^4^Center for Cancer Research, University of Tennessee Health Science Center, Memphis, TN, United States; ^5^Division of Gynecologic Oncology, National Hospital Organization, Hokkaido Cancer Center (NHO), Sapporo, Japan

**Keywords:** NEAT1, nuclear paraspeckle assembly transcript 1, long non-coding RNA, cancer diagnosis, cancer treatment, EMT, microRNA

## Abstract

The nuclear paraspeckle assembly transcript 1 (NEAT1, a long non-coding RNA) is frequently overexpressed in human tumors, and higher NEAT1 expression is correlated with worse survival in cancer patients. NEAT1 drives tumor initiation and progression by modulating the expression of genes involved in the regulation of tumor cell growth, migration, invasion, metastasis, epithelial-to-mesenchymal transition, stem cell-like phenotype, chemoresistance and radioresistance, indicating the potential for NEAT1 to be a novel diagnostic biomarker and therapeutic target. Mechanistically, NEAT1 functions as a scaffold RNA molecule by interacting with EZH2 (a subunit of the polycomb repressive complex) to influence the expression of downstream effectors of EZH2, it also acts as a microRNA (miRNA) sponge to suppress the interactions between miRNAs and target mRNAs, and affects the expression of miR-129 by promoting the DNA methylation of the miR-129 promoter region. Knockdown of NEAT1 via small interfering RNA or short hairpin RNA inhibits the malignant behavior of tumor cells. In this review, we highlight the latest insights into the expression pattern, biological roles and mechanisms underlying the function and regulation of NEAT1 in tumors, and especially focus on its clinical implication as a new diagnostic biomarker and an attractive therapeutic target for cancers.

## Introduction

Although most of the genome (around 80%) is actively transcribed into RNA, < 2% is actually translated into functional proteins (ENCODE Project Consortium, 2012), suggesting that the dominant fraction of the transcriptome consists of non-coding RNAs (ncRNAs). NcRNAs can be classified into small ncRNAs (< 200 nt) or long ncRNAs (>200 nt). One class of small ncRNAs, microRNAs (miRNAs), bind to the 3′-untranslated region of the target gene mRNA, thereby repressing target mRNA translation or inducing mRNA degradation. In contrast to well-studied miRNAs, the function and mechanism of long ncRNAs (lncRNAs) in human cancers are poorly characterized.

## Categories of lncRNAs

**Figure 1:**
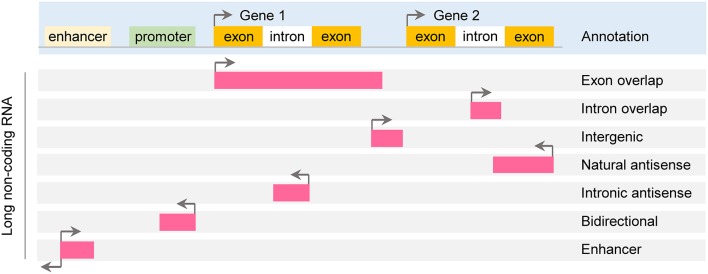
Classification of long noncoding RNA. Long non-coding RNAs (lncRNAs) are roughly classified based on their position relative to protein-coding genes: exon or intron sense-overlapping lncRNAs, intergenic lncRNAs, antisense lncRNAs, bidirectional lncRNAs and enhancer lncRNAs.

According to genomic location, lncRNAs can be further subdivided into exon or intron sense-overlapping lncRNAs, intergenic lncRNAs, antisense lncRNAs, bidirectional lncRNAs and enhancer lncRNAs (Esteller, 2011; Thum and Condorelli, 2015; Figure 1).

## Functions and mechanisms of lncRNAs

The function of lncRNA depends on their subcellular location (Chen L. L., 2016). Up to 30% of lncRNAs are found exclusively in the nucleus, ~15% are found exclusively in the cytoplasm, while the remaining lncRNAs are present in both nucleus and cytoplasm (Kapranov et al., 2007). In the nucleus, lncRNAs are important transcriptional and epigenetic modulators of nuclear functions, whereas cytoplasmic lncRNAs target mRNA transcripts and modulate mRNA stability and translation (Mercer and Mattick, 2013).

**Figure 2:**
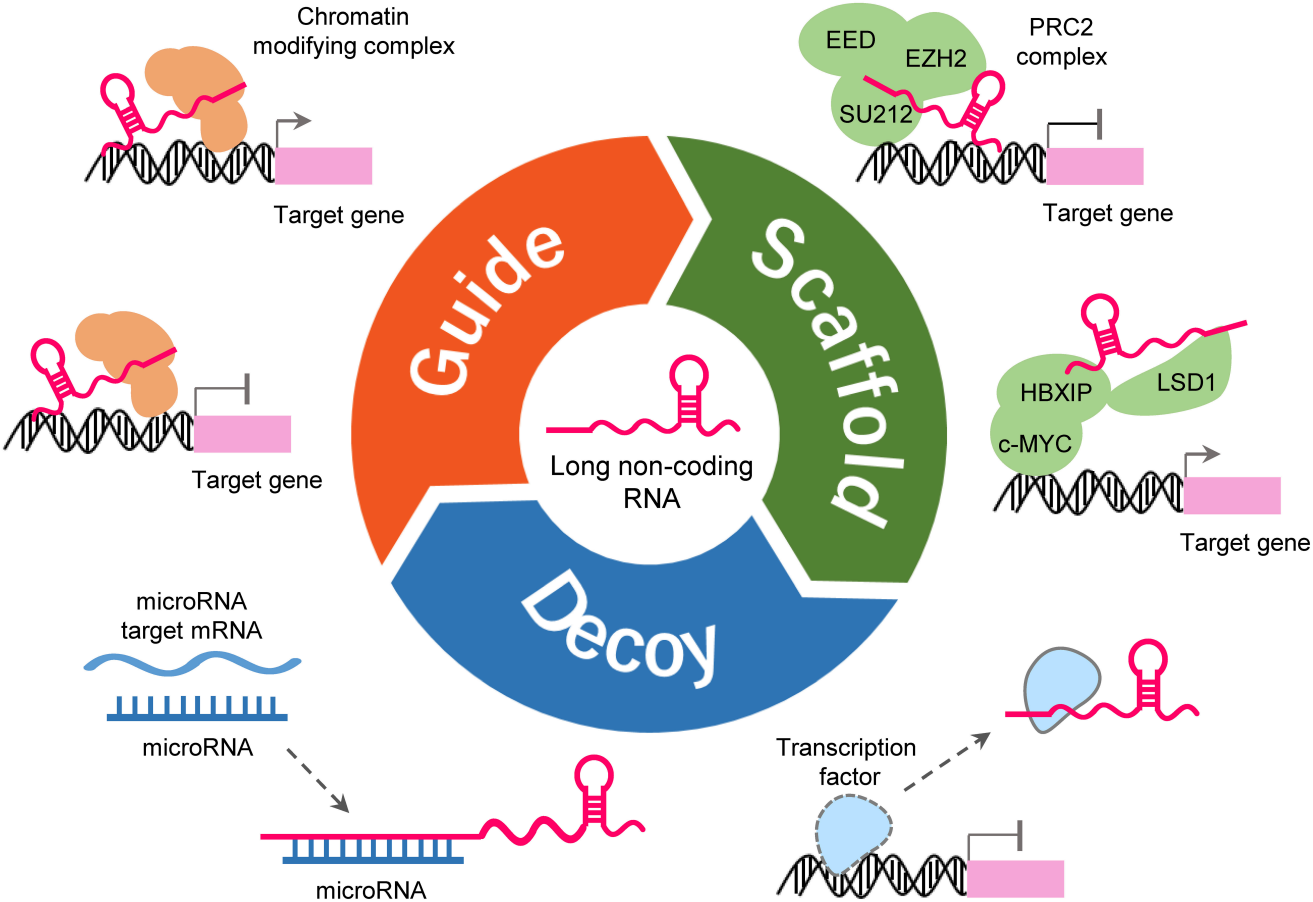
Functions and mechanisms of lncRNA. LncRNA can guide transcription factors to specific genomic locations for the regulation of gene expression **(upper-left)**. LncRNA works as a scaffold to facilitate the assembling of chromatin remodeling complexes **(upper-right)**. LncRNA can also serve as a sponge to titrate miRNAs out from their mRNA targets **(lower-left)**. LncRNA can bind to transcription factors or other proteins as a decoy and sequester them away from chromatin **(lower-right)**.

LncRNAs could form complex secondary and tertiary structures, which are proposed to provide multiple binding sites for other molecules (Liu et al., 2017b). LncRNAs regulates the expression of target genes by interacting with other molecules such as protein, DNA and RNA (Figure 2). Some lncRNAs can guide site-specific recruitment of transcriptional activators or suppressors to genomic sites and regulate gene expression (Mercer et al., 2009; Tripathi et al., 2010; Kotake et al., 2011; Wang and Chang, 2011; Li Y. et al., 2016; Xie et al., 2016). A number of lncRNAs may act as scaffolding proteins by recruiting chromatin remodeling complexes, including the polycomb repressive complex 1 (PRC1) and PRC2, to silence target-specific genes (Mercer et al., 2009; Tripathi et al., 2010; Kotake et al., 2011; Wang and Chang, 2011; Li Y. et al., 2016; Xie et al., 2016). Furthermore, lncRNAs can scaffold HBXIP and LSD1 to form a complex that activates transcription of c-Myc target genes (Mercer et al., 2009; Tripathi et al., 2010; Kotake et al., 2011; Wang and Chang, 2011; Li Y. et al., 2016; Xie et al., 2016). lncRNAs can bind to transcription factors as decoys to sequester them away from their targets, thereby affecting gene transcription (Mercer et al., 2009; Tripathi et al., 2010; Kotake et al., 2011; Wang and Chang, 2011; Li Y. et al., 2016; Xie et al., 2016). LncRNAs may serve as molecular sponges by harboring binding sites for miRNAs and sequester them away from their mRNA targets (Mercer et al., 2009; Tripathi et al., 2010; Kotake et al., 2011; Wang and Chang, 2011; Li Y. et al., 2016; Xie et al., 2016). Certain LncRNAs can regulate RNA splicing either by interacting with splicing factors or by binding the splicing junctions of pre-mRNA (Mercer et al., 2009; Tripathi et al., 2010; Kotake et al., 2011; Wang and Chang, 2011; Li Y. et al., 2016; Xie et al., 2016).

## The roles of lncRNAs in cancer

Increasing number of studies have shown that lncRNAs are either up- or down-regulated in cancers. Some lncRNAs are expressed in a cell type-specific manner during differentiation and in certain cancers, whereas several lncRNAs such as MALAT1 are widely overexpressed in various cancers (Prensner and Chinnaiyan, 2011; Wang Y. et al., 2016). Their dysregulation is associated with tumorigenesis, cancer progression, metastasis and prognosis of various tumors (Schmitt and Chang, 2016), suggesting that lncRNAs may have roles as potential biomarkers for cancer. Actually, lncRNAs have been identified as prognostic and diagnostic biomarkers for lymph node metastasis and distant metastasis in early-stage cancer (Chen J. et al., 2016). Furthermore, extracellular lncRNAs can be stable and detectable in bodily fluids (such as blood and urine), thus the levels of circulating lncRNAs can be considered as promising non-invasive biomarkers over conventional biomarkers (Shi et al., 2016).

Through the mechanisms mentioned in Figure 2, lncRNAs exert their oncogenic or tumor suppressive functions in human tumors and they are key regulators of pathways involving all hallmarks of cancer (Prensner and Chinnaiyan, 2011; Schmitt and Chang, 2016; Wang Y. et al., 2016). Due to the massive involvement of lncRNAs in the mechanistic, functional and translational aspects of cancer biology, they have been explored as therapeutic targets. For example, aberrant expression of the lncRNA H19 occurs in ovarian cancer and other types of cancers (Yoshimura et al., 2018). The toxin vector DTA-H19 is a plasmid that expresses the diphtheria toxin A chain under the control of the *H19* gene regulatory sequences (Mizrahi et al., 2009). The intra-tumoral injection of DTA-H19 caused significant inhibition of tumor growth in ovarian cancer xenograft models (Mizrahi et al., 2009). The safety, tolerability, and efficacy of DTA-H19 have been verified in a phase 1/2a clinical trial for the treatment of H19-overexpressing bladder cancer (Sidi et al., 2008).

Recent studies have demonstrated a complicated interplay and cross-regulation among different species of non-coding RNAs (Deng et al., 2016). LncRNAs and miRNAs can directly interact with and regulate each other through at least four mechanisms (Deng et al., 2016): (i) miRNA can decrease the abundance of lncRNA by reducing its stability (Yoon et al., 2012); (ii) lncRNAs can serve as sponges or decoys for miRNAs to decrease the available levels of miRNAs (Cesana et al., 2011); (iii) lncRNAs may compete with miRNAs for binding to mRNAs (Faghihi et al., 2010); and (iv) lncRNAs generate miRNAs from their exons and introns (Cesana et al., 2011).

## NEAT1, a novel player in tumor

**Figure 3:**
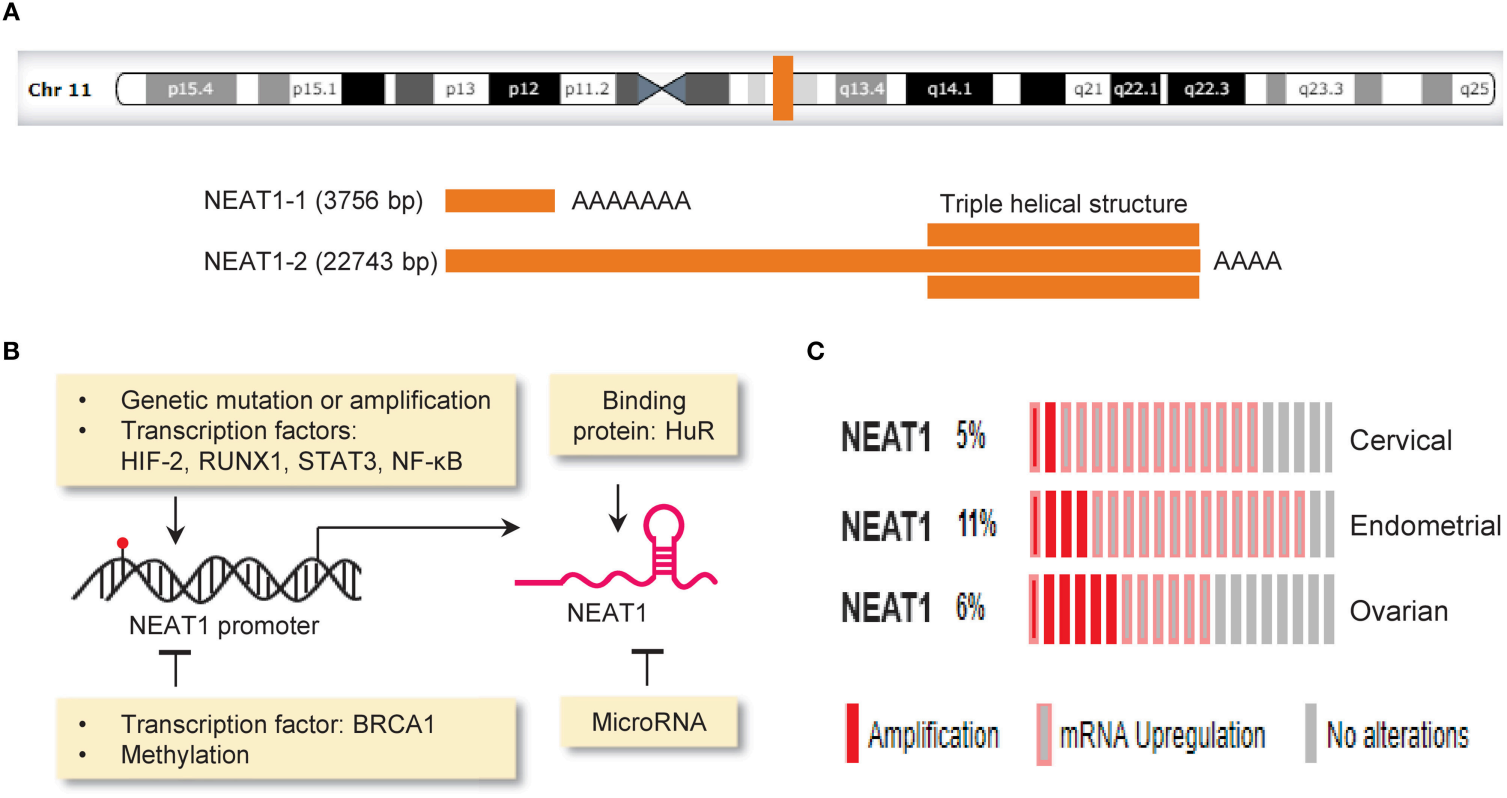
Mechanisms driving aberrant NEAT1 expression in cancer. **(A)** Genomic locus of the lncRNA NEAT1 on chromosome 11q13.1 (upper panel).The structure of NEAT1-1 and NEAT1-2 bottom panel). Two isoforms of NEAT1 (3,756 bp NEAT1-1 and 22,743 bp NEAT1-2) are transcribed from the same locus. NEAT1-2 possesses a unique triple helical structure at the 3′ end. **(B)** The expression of NEAT1 can be regulated by gene mutation (red), copy-number changes, transcription factors, DNA methylation, miRNA and RNA-binding protein. **(C)** Genetic alterations (amplification and RNA upregulation) of NEAT1 in samples of cervical, endometrial and ovarian cancer. The Cancer Genome Atlas (TCGA) datasets were retrieved in the cBioPortal database (www.cbioportal.org). Unaltered cases (gray) were not shown.

Nuclear paraspeckle assembly transcript 1 (NEAT1, a lncRNA) is transcribed from the familial tumor syndrome multiple endocrine neoplasia (MEN) type 1 loci on chromosome 11q13.1 and encodes two transcriptional variants, NEAT1-1 (3756 bp) and NEAT1-2 (22,743 bp) (Bond and Fox, 2009; Figure 3A). NEAT1 is enriched in the nucleus but also found in the cytoplasm (van Heesch et al., 2014). NEAT1 appears to be dispensable for normal embryonic development and adult life, as mice lacking NEAT1 develop normally (Nakagawa et al., 2011). However, another study reported that genetic ablation of NEAT1 resulted in aberrant mammary gland morphogenesis and lactation defects (Standaert et al., 2014). Whether the loss of NEAT1 is compatible with normal cell viability and normal development should be further evaluated.

NEAT1 displays typical characteristics of cancer drivers, because it is responsible for tumor initiation and progression, and its frequent dysregulation in cancers correlates with clinical features such as metastasis, recurrence rate and patient survival (Lanzós et al., 2017).

Increasing evidence suggested that NEAT1 is overexpressed in many solid tumors, including non-small cell lung cancer (Pan et al., 2015; Sun et al., 2016), ovarian cancer (Kim et al., 2010; Chen Z. J. et al., 2016), cervical cancer (Han et al., 2017), hepatocellular carcinoma (Guo et al., 2015; Liu Z. et al., 2017), colorectal cancer (Li et al., 2015; Peng et al., 2017), gastric cancer (Ma et al., 2016), esophageal squamous cell carcinoma (Chen et al., 2015), endometrial cancer (Li Z. et al., 2016; Wang J. et al., 2017), cholangiocarcinoma (Zhang C. et al., 2018), laryngeal squamous cell cancer (Wang P. et al., 2016), pancreatic cancer (Huang et al., 2017), thyroid carcinoma (Li J. H et al., 2017), oral squamous cell carcinoma (Huang et al., 2018), nasopharyngeal carcinoma (Cheng and Guo, 2017; Liu F. et al., 2018), osteosarcoma (Wang H. et al., 2017; Hu et al., 2018), breast cancer (Zhang et al., 2017; Zhao et al., 2017), glioma (Zhen et al., 2016) and renal cell carcinoma (Liu et al., 2017a; Ning et al., 2017). However, its expression was reduced in acute promyelocytic leukemia (Zeng et al., 2014), indicating that the role of NEAT1 may vary with cancer types.

The overexpression of NEAT1 was significantly associated with poor overall survival in non-small cell lung cancer (Pan et al., 2015), ovarian cancer (Chen Z. J. et al., 2016), cervical cancer (Han et al., 2017), colorectal cancer (Li et al., 2015; Peng et al., 2017), hepatocellular carcinoma (Liu Z. et al., 2017), esophageal squamous cell carcinoma (Chen et al., 2015), pancreatic cancer (Huang et al., 2017), oral squamous cell carcinoma (Huang et al., 2018), breast cancer (Zhao et al., 2017), gastric cancer (Fu et al., 2016), breast cancer (Zhang et al., 2017), and renal cell carcinoma (Ning et al., 2017). Moreover, higher expression levels of NEAT1 were positively correlated with cancer stage and metastasis in endometrial cancer (Li Z. et al., 2016), ovarian cancer (Chen Z. J. et al., 2016), non-small cell lung cancer (Pan et al., 2015; Sun et al., 2017), hepatocellular carcinoma (Sun et al., 2016), esophageal squamous cell carcinoma (Chen et al., 2015), laryngeal squamous cell cancer (Wang P. et al., 2016), breast cancer (Zhao et al., 2017), gastric cancer (Fu et al., 2016), and osteosarcoma (Hu et al., 2018).

## Mechanisms of NEAT1 dysregulation in cancer

The expression of NEAT1 in cancer cells is controlled by the following mechanisms: genetic alterations (such as copy number gain and gene mutation), transcription factors, DNA methylation, miRNAs and RNA-binding protein (Figure 3B).

The Cancer Genome Atlas (TCGA, a large-scale cancer genomics project) has revealed molecular alterations such as gene mutations, copy-number changes, upregulation or downregulation of mRNA and lncRNA across diverse tumor types. A comprehensive study analyzing lncRNA alterations in the TCGA datasets covering 5,860 tumor samples from 13 cancer types revealed that on average 13.16% of lncRNAs underwent copy number gains and 13.53% of lncRNAs underwent copy number loss (Yan et al., 2015). Here, we used the TCGA cervical, endometrial and ovarian cancer datasets to annotate genetic alterations of NEAT1 in these gynecological cancers via the cBioPortal online application (http://www.cbioportal.org). Consistent with previous observations (Kim et al., 2010; Chen Z. J. et al., 2016; Li Z. et al., 2016; Han et al., 2017; Wang J. et al., 2017), we found that genetic alterations occur in 5–11% of cervical, endometrial and ovarian cancers, and the predominant alterations were amplification and RNA upregulation (Figure 3C). The presence of copy number gain in *NEAT1* gene might provide a possible explanation for high NEAT1 expression in these cancers. In addition, deep sequencing analysis of breast cancers has indicated mutations in the *NEAT1* promoter region (Rheinbay et al., 2017). Another group also found an elevation of mutations in the *NEAT1* promoter in renal cell carcinoma, and these mutations were associated with increased NEAT1 expression and unfavorable patient survival (Li S. et al., 2017). Although the functional impact of these mutations is unknown, mutations that occur in the *NEAT1* promoter might affect the binding of a protein to the *NEAT1* promoter and alter its expression (Rheinbay et al., 2017).

Several studies reported that transcription factors such as hypoxia-inducible factor (HIF)-2 and RUNX1 bind to the locus of *NEAT1* and induce its expression in breast cancer (Choudhry et al., 2015; Barutcu et al., 2016). STAT3 and NF-κB, two downstream effectors of EGFR signaling, could bind to and activate the *NEAT1* promoter in glioblastoma (Chen et al., 2018). On the other hand, BRCA1 inhibits NEAT1 expression through binding to its genomic binding site upstream of the *NEAT1* gene in breast cancer (Lo et al., 2016). At the post-transcriptional level, NEAT1 is physically associated with and stabilized by an RNA-binding protein HuR (Chai et al., 2016).

Epigenetic mechanisms such as aberrant DNA methylation and miRNA dysregulation account for the aberrant expression of lncRNAs in tumors (Choudhry et al., 2015; Yan et al., 2015). The expression of NEAT1 was increased by the treatment with 5-AZA in hepatocellular carcinoma cells, indicating that DNA methylation is an important determinant of NEAT1 expression (Fang et al., 2017). Numerous miRNAs that directly target lncRNAs have identified in tumor cells (Braconi et al., 2011), and miRNAs that directly interact with NEAT1 will be discussed below. How epigenetic mechanisms (such as histone modifications) contribute to the transcriptional control of NEAT1 expression warrants further investigation.

## NEAT1 controls cancer initiation and progression

**Figure 4:**
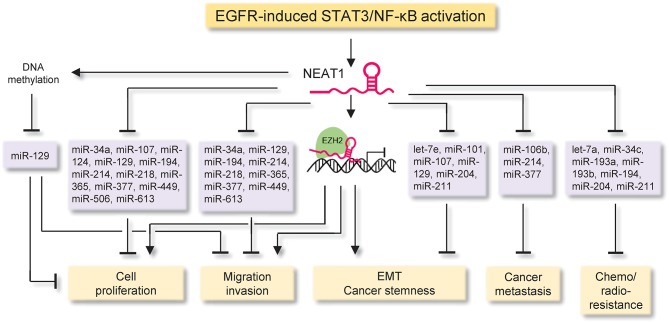
The proposed mechanisms of NEAT1 action in cancer. The diagram illustrates signaling events upstream and downstream of NEAT1 in cancer. The EGFR-STAT3/NF-κB signaling pathway is a critical triggering mechanism upstream of NEAT1, which inhibits cell cycle arrest and apoptosis, but promotes migration, invasion, metastasis, epithelial-to-mesenchymal transition, stem cell-like phenotype, chemoresistance and radioresistance, through at least three major mechanisms: (i) NEAT1 functions as a scaffold RNA molecule by interacting with EZH2 (a subunit of the polycomb repressive complex) to influence the expression of downstream effectors of EZH2, (ii) NEAT1 acts as a miRNA sponge to antagonize the interactions between multiple tumor suppressor miRNAs and target mRNAs, and (iii) NEAT1 suppresses the expression of miR-129 by promoting the DNA methylation of the miR-129 promoter.

NEAT1 inhibits cell cycle arrest and apoptosis, but promotes migration, invasion, metastasis, epithelial-to-mesenchymal transition (EMT), stem cell-like phenotype, chemoresistance and radioresistance, through at least three different molecular mechanisms (Figure 4 and Table 1): (i) NEAT1 functions as a scaffold RNA molecule by interacting with EZH2 (a subunit of the polycomb repressive complex) to influence the expression of downstream effectors of EZH2, (ii) NEAT1 acts as miRNA sponges to antagonize the interactions between multiple tumor suppressor miRNAs and target mRNAs, and (iii) NEAT1 suppresses the expression of miR-129 by promoting the DNA methylation of the miR-129 promoter region.

**Table 1:** NEAT1 is a key regulator of cancer initiation and progression.

**Cancer type**	**Function**	**Cell lines**	**Assay methods**	**Isoforms**	**Molecular mechanisms**	**References**
Non-small cell lung cancer	Oncogene	A549, SPC-A1, H1299, 95D, SK-MES-1, NCI-H520	Colony formation, CCK8, migration, invasion, in vivo tumor formation assay	NA	Functioning as a competing endogenous RNA for miR-377	(Sun et al., 2016)
Non-small cell lung cancer	Oncogene	A549, H1299, SPCA1, H358	CCK8, migration, invasion assay	NA	Activating the Wnt/β-catenin signaling pathway	(Sun et al., 2017)
Non-small cell lung cancer	Oncogene	A549, H460, H1299	Flow cytometry analysis	NA	Activating the Wnt/β-catenin signaling pathway and EMT-related pathway	(Jiang et al., 2018)
Cervical cancer	Oncogene	HeLa, SiHa	MTT, Flow cytometry analysis, Radiation sensitivity assay	NA	Functioning as a competing endogenous RNA for miR-193	(Han et al., 2017)
Colorectal Cancer	Oncogene	HCT116, LoVo, HT29, SW480	MTT, Flow cytometry analysis,	NA	Activating the AKT pathway	(Peng et al., 2017)
Colorectal Cancer	Oncogene or tumor suppressor	HCT116, LoVo	CCK8, invasion assay	NEAT1-1 or NEAT-2	NA	(Wu et al., 2015)
Esophageal squamous cell carcinoma	Oncogene	KYSE140, KYSE150	CCK8, Colony formation, migration, invasion assay	NA	NA	Chen et al., 2015
Endometrial cancer	Oncogene	HEC-59, KLE, AN3CA, HEC-1-A, HEC-1-B	CCK8, Colony formation, Cell cycle and cell apoptosis analysis, migration, invasion assay	NA	Up-regulating the expression of c-Myc, IFG1, MMP-2 and MMP-7	Li Z. et al., 2016
Endometrial cancer	Oncogene	HEC-1A	WST-1, migration, invasion assay	NA	Functioning as a competing endogenous RNA for miR-214	Wang J. et al., 2017
Cholangiocarcinoma	Oncogene	QBC939, RBE, HuCCT1, TFK1	CCK8, Colony formation, in vivo tumor formation assay	NA	Repressing E-cadherin transcription through binding with EZH2	(Zhang C. et al., 2018)
Laryngeal squamous cell cancer	oncogene	Hep-2	CCK8, Wound-healing, invasion assay, cell apoptosis analysis, in vivo tumor formation assay	NA	Functioning as a competing endogenous RNA for miR-107	Wang P. et al., 2016
Pancreatic cancer	Oncogene	AsPC-1, BxPC-3, SW1990, PANC-1	CCK8 assay, Cell cycle and cell apoptosis analysis	NA	Functioning as a competing endogenous RNA for miR-506	(Huang et al., 2017)
Thyroid carcinoma	Oncogene	TPC-1	CCK8, migration, invasion assay, in vivo tumor formation assay	NA	Functioning as a competing endogenous RNA for miR-214	Li J. H et al., 2017
Oral squamous cell carcinoma	Oncogene	Unknown	CCK8 assay, Cell cycle and cell apoptosis analysis	NA	Functioning as a competing endogenous RNA for miR-365	(Huang et al., 2018)
Nasopharyngeal carcinoma	Oncogene	CNE1, CNE2, SUNE1, 6-10B, SUNE2	CCK8, Colony formation assay, cell apoptosis analysis	NA	Functioning as a competing endogenous RNA for miR-124	(Cheng and Guo, 2017)
Nasopharyngeal carcinoma	Oncogene	CNE-2, HONE-1, 5-8F, SUNE-1	Colony formation, apoptosis assay	NA	Functioning as a competing endogenous RNA for miR-204	(Lu et al., 2016)
Nasopharyngeal carcinoma	Oncogene	5-8F, CNE1, CNE2, S26, HNE1, SUNE1, HONE1	MTT, Drug resistance, cell apoptosis, wound healing assay	NA	Functioning as a competing endogenous RNA for miR-101	(Wang H. et al., 2017)
Osteosarcoma	Oncogene	MG63, U2OS	MTT, invasion assay, cell apoptosis analysis	NA	Functioning as a competing endogenous RNA for miR-194	Wang Y. et al., 2017
Osteosarcoma	Oncogene	MG63, 143B, HOS, Saos2	CCK8 assay, cell apoptosis analysis, in vivo tumor formation assay	NA	Functioning as a competing endogenous RNA for miR-34c	(Hu et al., 2018)
Breast cancer	Oncogene	Unknown	CCK8, colony formation, invasion assay	NA	Functioning as a competing endogenous RNA for miR-218	(Zhao et al., 2017)
Breast cancer	Oncogene	MCF-7, MDA-MB-231, MDAMB-468, T47D, BT-547	MTT, wound Healing assay	NA	Activating the β-catenin signaling, and triggering EMT	(Zhang et al., 2017)
Breast cancer	Oncogene	MCF-7, SKBR3, MDA-MB-453, T47D, DU4475	MTT, BrdU incorporation assay	NA	Functioning as a competing endogenous RNA for miR-101	(Qian et al., 2017)
Breast cancer	Oncogene	MCF-7, MDA-MB-231, T47D, ZR-75-1	Migration, invasion, in vivo tumor formation and metastasis assay	NA	Functioning as a competing endogenous RNA for miR-211	Li X. et al., 2017
Breast cancer	Oncogene	MCF-7, HCC1937	Migration, invasion, Soft agar colony formation assay, in vivo tumor formation assay	NA	Silencing miR-129-5p expression by promoting the DNA methylation of the CpG island in the miR-129 gene	(Lo et al., 2016)
Glioma	Oncogene	U87, U373, U251	MTT, migration, invasion assay, cell apoptosis analysis, in vivo tumor formation assay	NA	Functioning as a competing endogenous RNA for miR-449b	(Zhen et al., 2016)
Glioma	Oncogene	U87, Glioma primary cultured cells	Sphere formation, Soft agar colony formation assay, cell cycle and cell apoptosis analysis	NA	Functioning as a competing endogenous RNA for miR-107	(Yang et al., 2017)
Clear cell renal cell carcinoma	Oncogene	Unknown	CCK8, migration, invasion assay, cell apoptosis analysis	NA	Inducing the EMT process	(Ning et al., 2017)
Renal cell carcinoma	Oncogene	ACHN, 786-O, A498, Caki-1	EdU, migration, invasion assay, cell cycle analysis	NA	Functioning as a competing endogenous RNA for miR-34c	(Liu et al., 2017a)
Acute promyelocytic leukemia	Tumor suppressor	NB4, NB4-R2, U937-PR9	Flow cytometry analysis	NEAT1-1 and NEAT1-2	NA	(Zeng et al., 2014)
Gastric cancer	Oncogene	NCI-N87, SGC-7901, MKN-45, AGS	Colony formation, migration, invasion assay	NA	Inducing the EMT process	(Fu et al., 2016)
Gastric cancer	Oncogene	MKN-45, BGC823, MGC803, SGC7901, AGS, MKN28	WST-1, migration assay	NA	NA	(Ma et al., 2016)
Glioblastoma	Oncogene	N5, N9, and N33 patient-derived cells	CCK8, colony formation, invasion assay, cell apoptosis assay, in vivo tumor formation assay	NA	Recruiting EZH2 to form the PRC2 complex and mediate the expression of EZH2 target genes	Chen et al., 2018
Hepatocellular carcinoma	Oncogene	MHCC97H, MHCC97L, SMCC7721, Huh7	MTT, invasion assay	NA	Functioning as a competing endogenous RNA for miR-613	Wang Z. et al., 2017
Thyroid cancer	Oncogene	NPA8, TPC-1, KAT-5	Colony formation, wound healing, invasion assay, in vivo tumor formation assay	NA	Functioning as a competing endogenous RNA for miR-129	(Zhang H. et al., 2018)
Thyroid cancer	Oncogene	K1, TPC-1	CCK8, wound healing, migration, invasion assay, cell apoptosis assay	NEAT1-2	Functioning as a competing endogenous RNA for miR-106b	(Sun et al., 2018)
Ovarian cancer	Oncogene	OVCAR3, SKOV3, HO8910, OV90	MTT, caspase-3 activity assay	NA	Functioning as a competing endogenous RNA for miR-34a	(Ding et al., 2017)
Ovarian cancer	Oncogene	SKOV3, HeyA-8	Drug resistance assay, cell apoptosis assay, in vivo tumor formation assay	NA	Functioning as a competing endogenous RNA for miR-194	(An et al., 2017)
Multiple myeloma	Oncogene	RPMI8226, JJN-3, U266, ANBL6, OPM-2, MM1S, MM1R	MTT, cell apoptosis assay	NA	Functioning as a competing endogenous RNA for miR-193a	(Wu and Wang, 2018)

*NA, Undetermined*.

In gastric cancer, laryngeal squamous cell cancer, pancreatic cancer, oral squamous cell carcinoma, nasopharyngeal carcinoma and breast cancer, knockdown of NEAT1 via small interfering RNA (siRNA) inhibited cell proliferation (Ma et al., 2016; Wang P. et al., 2016; Cheng and Guo, 2017; Huang et al., 2017, 2018; Qian et al., 2017). Similarly, the inhibition of NEAT1 via siRNA reduced cancer cell proliferation, migration and invasion in esophageal squamous cell carcinoma, endometrial cancer, glioma, hepatocellular carcinoma and renal cell carcinoma (Chen et al., 2015; Li Z. et al., 2016; Zhen et al., 2016; Ning et al., 2017; Wang Z. et al., 2017). Furthermore, short hairpin RNA (shRNA)-mediated silencing of NEAT1 significantly impaired cell proliferation, migration and invasion in cholangiocarcinoma (Zhang C. et al., 2018). Conversely, forced expression of NEAT1 enhanced cell growth, migration and invasion in endometrial cancer, papillary thyroid cancer, nasopharyngeal carcinoma, ovarian cancer, non-small cell lung cancer and breast cancer (Li Z. et al., 2016; Sun et al., 2016; Cheng and Guo, 2017; Ding et al., 2017; Wang J. et al., 2017; Zhao et al., 2017; Zhang H. et al., 2018).

EMT is a complex process in which epithelial cells acquire the characteristics of invasive mesenchymal cells and has been shown to contribute to tumorigenesis, invasion, metastasis, resistance to conventional chemotherapy, radiotherapy and small-molecule-targeted therapy (Dong et al., 2016; Nieto et al., 2016; Huo et al., 2017). Cancer stem cells (CSCs) are a class of pluripotent cells that possess a capacity for self-renewal and are resistant to chemotherapy and radiotherapy (Ayob and Ramasamy, 2018). Previous studies have established a link between EMT and CSC formation (Mani et al., 2008; Dong et al., 2014). NEAT1 overexpression promotes EMT and invasion in breast cancer, renal cell carcinoma, renal cell carcinoma, hepatoblastoma and nasopharyngeal carcinoma (Lu et al., 2016; Fu et al., 2017; Liu et al., 2017a; Li W. et al., 2017; Ning et al., 2017; Zhang et al., 2017; Zheng et al., 2018). Importantly, NEAT1 was found to be overexpressed in CD133^+^ glioma stem cells and knockdown of NEAT1 by siRNA reduced the ability of these cells to form colonies in soft agar (Yang et al., 2017). Glioma stem cells transfected with NEAT1 shRNA exhibited weaker proliferation, migration and invasion than that of those cells transfected with control shRNA (Gong et al., 2016). In sphere-forming cells generated from certain non-small cell lung cancer cell lines, downregulation of NEAT1 resulted in a significant decrease in the expression of CSC markers (CD133, CD44, ABCG2, Sox2, Nanog, and Oct-4) (Jiang et al., 2018). Not surprisingly, NEAT1 silencing by siRNA sensitized tumor cells to anti-cancer drugs such as sorafenib (Liu et al., 2017a), cisplatin (Hu et al., 2018; Liu F. et al., 2018), dexamethasone (Wu and Wang, 2018), and paclitaxel (An et al., 2017). shRNA-mediated reduction of NEAT1 enhanced the *in vitro* radiosensitivity of cancer cell lines to radiation therapy (Lu et al., 2016; Han et al., 2017).

NEAT1-1 and NEAT1-2 appear to have distinct roles in regulating the phenotypes of cancer cells. For example, in colorectal cancer cell lines, knockdown of NEAT1-1 could inhibit cell invasion and proliferation, whereas knockdown of NEAT1-2 promoted cell growth (Wu et al., 2015). The same study showed that expression of NEAT1-1 was significantly higher in liver metastatic lesions compared with adjacent normal colorectal tissues and primary colorectal cancer tissues (Wu et al., 2015). These findings suggested that NAT1-1 may act as a carcinogenic factor, while NEAT1-2 may be a tumor suppressor in colorectal cancer. It appears that the expression of NEAT1 isoforms is regulated in a cell type-specific manner. For example, in adult mouse tissues, NEAT1-1 is expressed in a broad range of cell types (Nakagawa et al., 2011). However, NEAT1-2 expression is largely restricted to the epithelial layers of digestive tissues (Nakagawa et al., 2011). The compositions of NEAT1 isoforms may vary in different cancer types. These dynamic changes in NEAT1 isoform expression may affect the main function of NEAT1 as an oncogene or a tumor suppressor. Therefore, elucidating the exact contribution of NEAT1 isoforms to the development of human tumors would be an exciting direction for future studies.

Mechanistically, NEAT1 recruits EZH2 to form the PRC2 complex and mediate the expression of EZH2 target genes, thereby promoting tumor cell growth and invasion in glioblastoma and cholangiocarcinoma (Chen et al., 2018; Zhang C. et al., 2018; Figure 4 and Table 1).

Additionally, by sponging a set of miRNAs, such as let-7a (Liu F. et al., 2018), let-7e (Gong et al., 2016), miR-34a (Ding et al., 2017; Liu et al., 2017a), miR-34c (Hu et al., 2018), miR-101 (Qian et al., 2017; Wang Y. et al., 2017), miR-106b (Sun et al., 2018), miR-107 (Wang P. et al., 2016; Yang et al., 2017), miR-124 (Chai et al., 2016; Cheng and Guo, 2017; Liu X. et al., 2018), miR-193a (Wu and Wang, 2018), miR-193b (Han et al., 2017), miR-194 (An et al., 2017; Wang H. et al., 2017), miR-204 (Lu et al., 2016), miR-211 (Li X. et al., 2017), miR-214 (Li J. H et al., 2017; Wang J. et al., 2017), miR-218 (Zhao et al., 2017), miR-365 (Huang et al., 2018), miR-377 (Sun et al., 2016), miR-449 (Zhen et al., 2016), miR-506 (Huang et al., 2017), and miR-613 (Wang Z. et al., 2017), NEAT1 could abolish miRNAs-mediated suppression of their target genes, therefore promoting tumor cell growth, migration, invasion, metastasis, EMT, stem cell-like phenotype, chemoresistance and radioresistance (Figure 4 and Table 1).

Changes in miRNA expression through altered DNA methylation affect tumor progression. NEAT1 was shown to silence the expression of miR-129 by increasing the DNA methylation level in its promoter region in breast cancer cells (Lo et al., 2016; Table 1).

Of note, NEAT1 overexpression induced the activity of the Wnt/β-catenin signaling, either by inhibiting the expression of miR-214 (Wang J. et al., 2017) or by interacting with EZH2 to reduce the expression of negative regulators of WNT/β-catenin signaling such as ICAT, GSK3B and Axin2 (Chen et al., 2018 Table 1).

## NEAT1 as a potential diagnostic biomarker of cancer

Cancer type- or subtype-specific expression patterns and the sensitive and inexpensive quantitative detection methods for ncRNAs make lncRNAs suitable as hopeful biomarkers for cancer diagnosis and prognosis (Brunner et al., 2012; Bijnsdorp et al., 2017). LncRNAs can exhibit different expression patterns between subtypes of the same cancer (Su et al., 2014). Several lncRNAs (such as PCA3, PCGEM1 and PCAT-1) are highly specific to prostate cancer (Crea et al., 2014) and have been used to identify primary tumors.

Although lncRNAs are commonly expressed at lower levels than protein-coding mRNAs, some lncRNAs (including NEAT1) exhibit moderate to high levels of expression in clinical samples. Moreover, lncRNAs can be detected in body fluids such as serum, plasma, urine and saliva of cancer patients (Chandra Gupta and Nandan Tripathi, 2017). The diagnostic value of NEAT1 was validated in a cohort of colorectal cancer patients, because the whole-blood NEAT1 expression was significantly increased in cancer patients than persons without cancer (Wang Y. et al., 2017). Therefore, NEAT1 may be possibly used as a biomarker for the existence of primary cancer. The same study also reported that the whole-blood expression of NEAT1-1 was significantly higher in colorectal cancer patients with distant metastasis than in those without metastasis (Wang Y. et al., 2017). Thus, the whole-blood NEAT1 expression might serve as a biomarker for prediction of distant metastasis.

## NEAT1 as a potential therapeutic target in cancer

**Figure 5:**
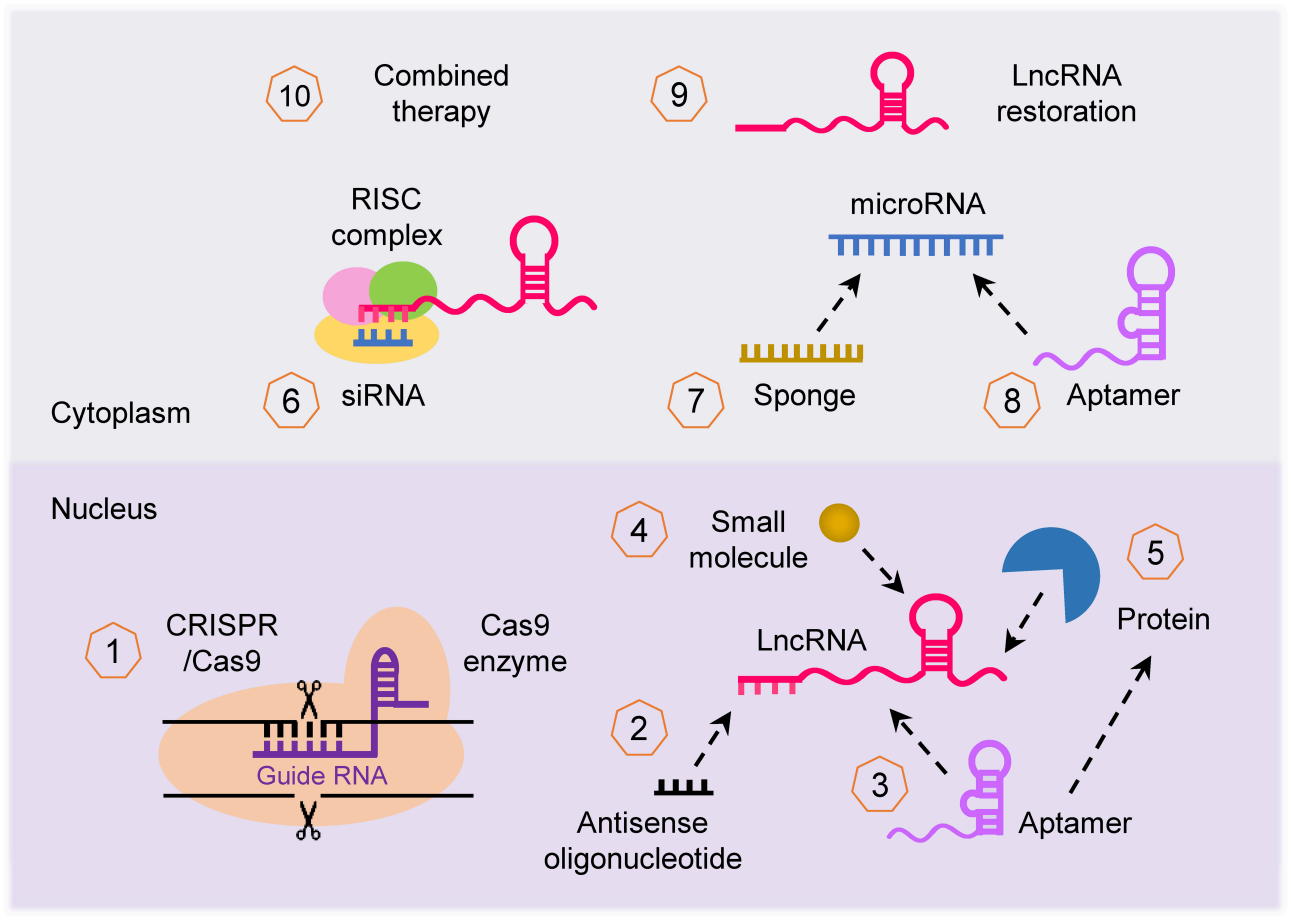
Therapeutic strategies to target lncRNA. Several approaches can be applied to target lncRNA in cancer for therapeutic purposes: (1) inhibition of lncRNA loci using the CRISPR/Cas-9 system, (2) knockdown of lncRNA transcript using antisense oligonucleotide, (3, 4) interfering with the binding of lncRNA with their binding partner (protein, DNA and RNA) with small synthetic molecule, peptide and aptamer, (5) repression of lncRNA that function through chromatin remodeling using selective PRC1/2 inhibitors, (6) silencing of lncRNA using specific siRNA, (7, 8) indirect targeting of lncRNA through modulating the function of miRNAs via miRNA mimics or anti-miRNA inhibitors such as aptamer, (9) restoring the expression of tumor suppressor lncRNA with synthetic lncRNA mimics, and (10) lncRNA can be applied for enhancing the effectiveness of other forms of therapy (such as chemotherapy and radiotherapy).

Given the importance of the lncRNA-mediated networks that broadly affect cancer imitation and progression, lncRNAs are of interest for developing novel therapeutics. LncRNAs can be targeted by multiple approaches (Parasramka et al., 2016; Worku et al., 2017; Arun et al., 2018; Figure 5): (i) CRISPR/Cas-9 system can be used to knockout lncRNA; (ii) lncRNA transcript can be knocked down using lncRNA-specific siRNA and antisense oligonucleotide (ASO); (iii) small synthetic molecules/peptides/aptamers can be designed to block and antagonize the binding of lncRNAs with their binding partners (such as protein, DNA, RNA, or other interacting complexes); (iv) selective PRC1/2 inhibitors such as GSK343 (Ihira et al., 2017) can be used to inhibit the activity of lncRNA that function through chromatin remodeling; (v) miRNA regulates the expression of lncRNA, thus restoration or blocking of a specific miRNA using miRNA mimics or anti-miRNA inhibitor (such as locked nucleic acid and miRNA sponge) can indirectly alter the expression of lncRNA; (vi) synthetic lncRNA mimics could be used to restore the expression of tumor suppressor lncRNA; and (vii) approaches to target lncRNAs can be used in combination with other therapies such as chemotherapy and radiotherapy to enhance their effectiveness. For example, targeted depletion of NEAT1 by siRNA (Li J. H et al., 2017) or shRNA (Zhang C. et al., 2018; Zhang H. et al., 2018) displayed a significant therapeutic effect *in vivo*. *In vivo* studies also confirmed that knockdown of NEAT1 sensitized tumor cells to cisplatin/paclitaxel- or radio-induced tumor regression (An et al., 2017; Han et al., 2017; Hu et al., 2018).

Apart from these strategies, targeting the upstream signaling pathways of NEAT1 or its binding partners would be another method to modulate NEAT1 expression or function in malignant tumors. Despite their potential side effects, blocking of NF-κB and STAT3 activity may represent a good approach to combat NEAT1-overexpressing tumors (Grivennikov and Karin, 2010; Liby et al., 2010). In addition, hypoxia is frequently observed in solid tumors and HIF-1/-2 has been potential targets for developing novel cancer therapeutics. A number of small molecule inhibitors of HIF-2 have been developed and might be used to downregulate the expression of NEAT1 (Yu et al., 2017). HuR is overexpressed in a wide variety of cancer types and stabilizes a large subset of mRNAs, which encode proteins implicated in tumor cell proliferation, survival, angiogenesis, invasion and metastasis (Kotta-Loizou et al., 2016). High-throughput screening methods have been established to identify small molecular molecules against HuR (Meisner et al., 2007). Thus, HuR inhibition may offer new conceptual routes to treat cancers expressing high levels of NEAT1.

## Perspectives and challenges

The current research on NEAT1 is still at a very early stage, but growing evidence has clearly identified NEAT1 as an attractive biomarker of cancer and an ideal candidate for lncRNA therapeutics. Although the broad implications of NEAT1 in cancers have been described, several key challenges associated with NEAT1 still exist: (i) The exact mechanisms for NEAT1-mediated carcinogenesis and metastasis remain elusive, although the interactions between PRC2 complex and miRNAs appears to be at least involved in these processes. High-throughput technologies such as ChIRP, PAR-CLIP and iCLIP have been used to reveal NEAT1-DNA or NEAT1-protein interactions (Hafner et al., 2010; Zhao et al., 2010; Chu et al., 2011; Tollervey et al., 2011; Yoon et al., 2014). (ii) The precise mechanisms by which the genetic and epigenetic factors contribute to NEAT1 dysregulation, as well as the downstream signaling pathways of NEAT1, are still far from clear. (iii) The *NEAT1* loci might be targeted for therapeutic purposes using CRISPR/Cas-9 genome editing technology. However, a challenge is the safety evaluation of CRISPR/Cas-9-based therapeutics, such as the risk of disease occurrence due to unwanted mutations, the immune response to the delivery system and other toxic side effects. Taken together, NEAT1 has the potential to provide a new biomarker for the diagnosis and monitoring of cancers and will need to be taken into consideration in the development of NEAT1-targeting therapeutics (possibly in combination with chemotherapy and radiotherapy).

## Author contributions

PD and HW provided direction. PD, YX, and HW wrote the manuscript. JY, SH, NK, and YT made significant revisions to the manuscript. All authors read and approved the final manuscript.

### Conflict of interest statement

The authors declare that the research was conducted in the absence of any commercial or financial relationships that could be construed as a potential conflict of interest.
